# 

**Published:** 1884-03

**Authors:** 


### THE CHICAGO MEDICAL JOURNAL & EXAMINER.

CHICAGO MEDICAL PRESS ASSOCIATION.

AUG. 2D

1875.

CHICAGO:

*PUBLISHED BY THE CHICAGO MEDICAL PRESS ASSOCIATION*

188 CLARK STREET.

☞ **Read the Notice of this Journal among Advertisements**

